# Characterization of Drug-Specific CD4^+^ T-Cells Reveals Possible Roles of HLA Class II in the Pathogenesis of Carbamazepine Hypersensitivity Reactions

**DOI:** 10.1021/acs.chemrestox.2c00414

**Published:** 2023-04-19

**Authors:** Kanoot Jaruthamsophon, Paul J. Thomson, Sean Hammond, Eunice Zhang, Ana Alfirevic, Chonlaphat Sukasem, Dean J. Naisbitt, Munir Pirmohamed

**Affiliations:** † Centre for Drug Safety Science, Department of Pharmacology and Therapeutics, Institute of Systems, Molecular and Integrative Biology, University of Liverpool, Liverpool L69 3GE, U.K.; ‡ Department of Pathology, Faculty of Medicine, Prince of Songkla University, Songkhla 90110, Thailand; § ApconiX, Alderley Park, Alderley Edge, Cheshire SK10 4TG, U.K.; ∥ Division of Pharmacogenomics and Personalized Medicine, Department of Pathology, Faculty of Medicine Ramathibodi Hospital, Mahidol University, Bangkok 10400, Thailand; ⊥ Laboratory for Pharmacogenomics, Somdech Phra Debaratana Medical Center (SDMC), Ramathibodi Hospital, Bangkok 10400, Thailand

## Abstract

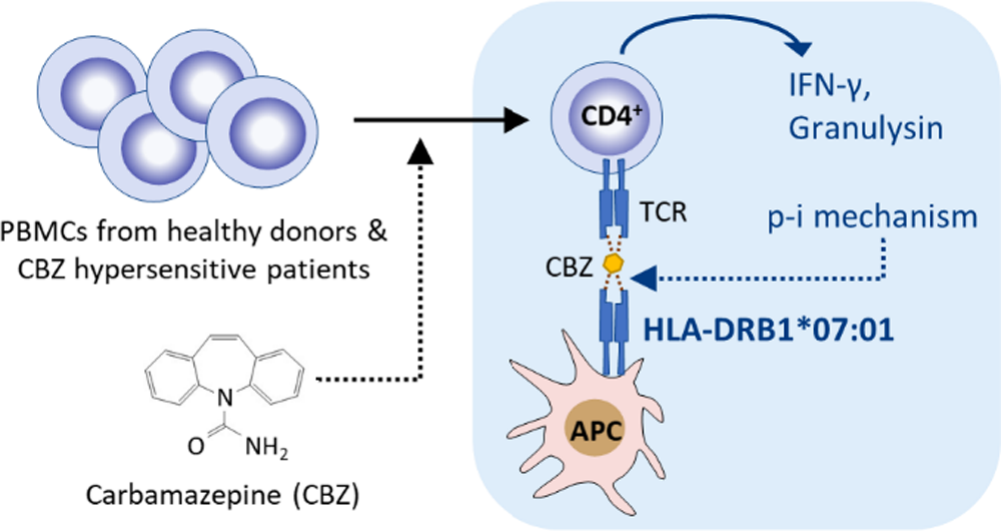

Carbamazepine (CBZ) is an aromatic anticonvulsant known to cause drug hypersensitivity reactions, which range in severity from relatively mild maculopapular exanthema to potentially fatal Stevens–Johnson syndrome and toxic epidermal necrolysis (SJS–TEN). These reactions are known to be associated with human leukocyte antigen (HLA) class I alleles, and CBZ interacts preferentially with the related HLA proteins to activate CD8^+^ T-cells. This study aimed to evaluate the contribution of HLA class II in the effector mechanism(s) of CBZ hypersensitivity. CBZ-specific T-cells clones were generated from two healthy donors and two hypersensitive patients with high-risk HLA class I markers. Phenotype, function, HLA allele restriction, response pathways, and cross-reactivity of CBZ-specific T-cells were assessed using flow cytometry, proliferation analysis, enzyme-linked immunosorbent spot, and enzyme-linked immunosorbent assay. The association between HLA class II allele restriction and CBZ hypersensitivity was reviewed using Allele Frequency Net Database. Forty-four polyclonal CD4^+^ CBZ-specific T-cell clones were generated and found to be restricted to HLA-DR, particularly HLA-DRB1*07:01. This CD4^+^-mediated response proceeded through a direct pharmacological interaction between CBZ and HLA-DR molecules. Similar to the CD8^+^ response, CBZ-stimulated CD4^+^ clones secreted granulysin, a key mediator of SJS–TEN. Our database review revealed an association between HLA-DRB1*07:01 and CBZ-induced SJS–TEN. These findings implicate HLA class II antigen presentation as an additional pathogenic factor for CBZ hypersensitivity reactions. Both HLA class II molecules and drug-responsive CD4^+^ T-cells should be evaluated further to gain better insights into the pathogenesis of drug hypersensitivity reactions.

## Introduction

Carbamazepine (CBZ) is an aromatic anticonvulsant known to cause hypersensitivity reactions. Various human leukocyte antigen (HLA) class I markers have been associated with a spectrum of drug reactions, ranging from a mild maculopapular exanthema (MPE) to severe cutaneous adverse reactions. A single HLA marker can be associated with more than one manifestation of hypersensitivity; for example, HLA-A*31:01 is known to be associated with CBZ-induced MPE, drug reaction with eosinophilia and systemic symptoms (DRESSs), Stevens–Johnson syndrome and toxic epidermal necrolysis (SJS–TEN).^1^ Conversely, a single reaction type can also be associated with multiple markers—this is seen with CBZ-induced SJS–TEN, which is associated with HLA-B*15:02 in Han Chinese,^2^HLA-B*15:11 in East Asians,^3,4^HLA-B*15:21 in Southeast Asians,^5−8^ and HLA-B*57:01 in Europeans.^9,10^ Functionally, these HLA molecules present the CBZ (and its metabolites) to CD8^+^ T-cells and cause drug-mediated responses via proliferation and secretion of cytotoxic mediators.^10−12^ However, in some instances, patients develop drug hypersensitivity reactions in the absence of any reported HLA class I risk alleles, indicating the contribution of other undiscovered pathogenic factors.

In addition to the HLA class I alleles, associations between other genetic markers and CBZ hypersensitivity have been demonstrated. HLA class II alleles were among the very first markers reported to be associated with CBZ hypersensitivity,^13^ but this was later described to be due to linkage disequilibrium with HLA class I alleles as part of a haplotype.^2,14,15^ However, these associations were inconsistent, especially in genome-wide association studies (GWASs) in which none of HLA class II markers reached genome-wide statistical significance.^16,17^ Interestingly, in functional studies, CBZ-responsive CD4^+^ and CD8^+^ T-cells have been generated in vitro from CBZ-hypersensitive patients and found to proliferate and secrete cytokines in the presence of the drug.^11,18,19^ It is, therefore, possible that class II HLA molecules play an important role in the pathogenesis of CBZ hypersensitivity, and that they have not been identified in GWASs because of the strict statistical definition (*P* values ≤ 5 × 10^–8^) used for genome-wide significance. Thus, we hypothesized that HLA class II markers could be an additional key pathogenic (and contributory) factor in CBZ hypersensitivity and that there could be multiple HLA class II markers with the capacity to present the CBZ molecule and elicit immune responses via a CD4^+^ T-cell-mediated mechanism. To test this hypothesis, we generated CBZ-responsive CD4^+^ T-cells using an in vitro T-cell cloning method and proceeded to characterize their phenotype and function.

## Experimental Procedures

### Human Subjects

Peripheral blood mononuclear cells (PBMCs) from two CBZ-naïve healthy donors (donor D1, D3 positive for HLA-A*31:01 and donor D2, D4 positive for HLA-B*15:02) and two CBZ hypersensitive patients (DRESSs patient P1 positive for HLA-A*31:01 and MPE patient P2 positive for HLA-B*57:01) were used for in vitro generation of CBZ-specific T-cell clones. P1 was previously characterized in our previous study.^11^ All donors and patients were recruited and obtained informed consent at the Royal Liverpool University Hospital, the University of Liverpool. Clinical information and HLA genotypes of each donor and patient are available in Supplementary Table S1. Approval for the investigations was obtained from the Liverpool Research Ethics committee and informed written consent was obtained from each donor.

### Generation and Characterization of CBZ-Specific T-Cell Clones

CBZ-specific T-cell clones were generated by T-cell cloning methodologies as previously described.^18^ Epstein–Barr Virus (EBV)-transformed B-cells were generated and used as antigen-presenting cells (APCs) to analyze for proliferation and cytokine release. Drug specificity of the T-cell clones was tested by proliferation analysis. The CD4/CD8 and T-cell receptor (TCR) Vβ phenotype of generated clones was assessed by flow cytometry. Interferon (IFN)-γ and granulysin release was measured by enzyme-linked immunosorbent spot (ELISpot) and enzyme-linked immunosorbent assay (ELISA), respectively. HLA restriction of CBZ-specific T-cell clones was evaluated by HLA blocking analyses and HLA mismatch analyses. HLA blocking analyses were performed using an anti-HLA class I antibody (W6/32, Abcam), anti-HLA class II antibody (6C6, Abcam), and a panel of anti-HLA-DP (B7/21), anti-HLA-DQ (SPV-L3), and anti-HLA-DR (L243, Abcam) antibody. The mechanism of binding of CBZ was determined by APC pulsing analysis and glutaraldehyde fixation analysis.^20^ Online database review was conducted using the Allele Frequency Net Database (AFND) to validate the HLA allele restriction result.^21^ Full details of methods are available as Supplementary Materials and Methods. The study protocol was approved by the Liverpool ethics committee.

### Statistical Analysis

Statistical analysis was performed using SigmaPlot 14.0. Figures were originally illustrated using SigmaPlot 14.0, Microsoft Excel, and Microsoft PowerPoint. CBZ-specific T-cell activation in APC mismatched analyses and anti-HLA blocking experiments were compared using the *t* test. Frequencies of in vitro drug-specific clones were compared using a chi-square test. All reported associations between HLA class II alleles and the CBZ hypersensitivity reactions were calculated using the chi-square test with Yate’s continuity correction or Fisher’s exact test (when more than 20% of expected values were less than 5). A *P* value of ≤0.05 was accepted as significant.

## Results

### Heterogeneous CD4^+^ CBZ-Specific T-Cell Clones Can Be Generated from Healthy Donors and Hypersensitive Individuals

**Figure 1:**
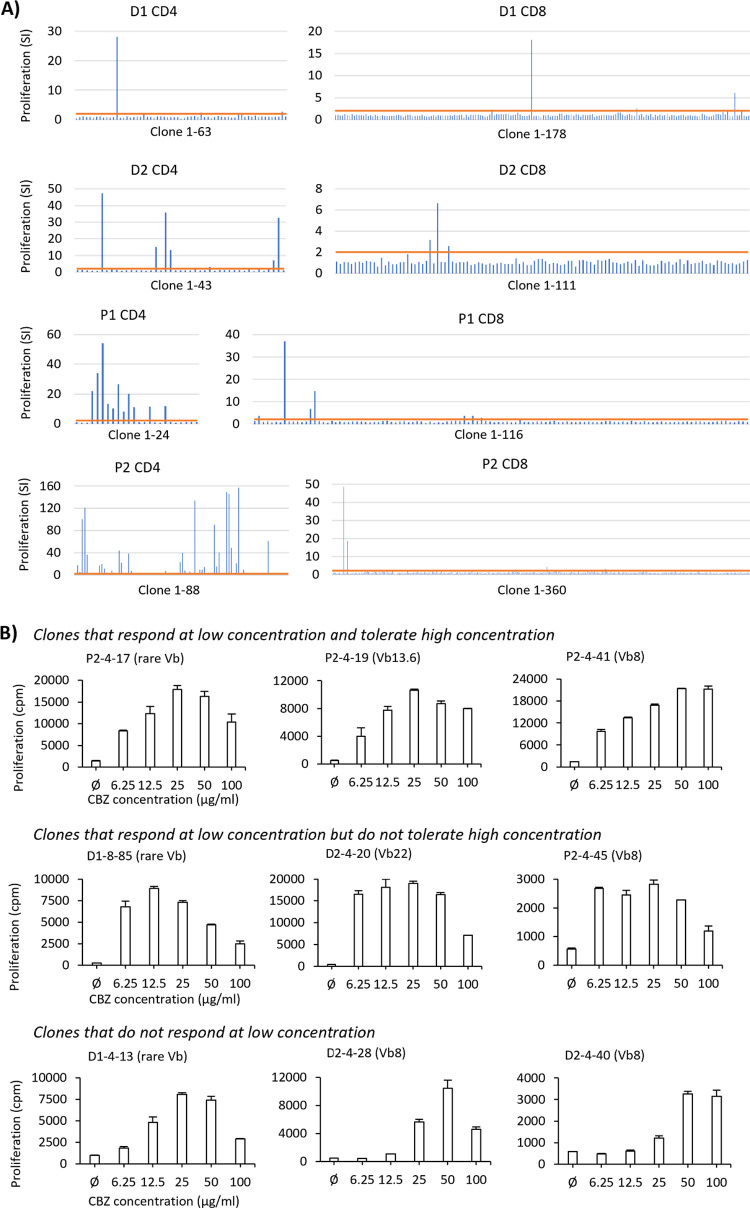
Characteristics of generated carbamazepine (CBZ)-specific T-cell clones revealed intra-individual variation. (A) T-cell clones (around 5 × 10^4^/well) expanded from CBZ-stimulated PBMCs were cultured in duplicate with autologous EBV-transformed B-cells (1 × 10^4^/well) in the presence and absence of CBZ (25 μg/mL). The proliferation of each T-cell clone was measured (in counts per minute; cpm) and the stimulation index was calculated (SI = cpm with drug/cpm without drug). Each vertical line represents the SI of each clone. Horizontal line represents SI = 2. (B) CBZ-specific clones from healthy donor D2 showed clonal variation in dose-dependent response patterns with CBZ. T-cell clones (5 × 10^4^/well) were cultured for 48 h at 37 °C, 5%CO_2_ in duplicate with autologous EBV-transformed B-cells (1 × 10^4^/well) in the presence and absence of graded concentration of CBZ (6.25–100 μg/mL). The proliferation activity was determined using the [^3^H]-thymidine incorporation assay. Data showed mean proliferation (cpm: counts per minute) and SEM.

**Figure 2:**
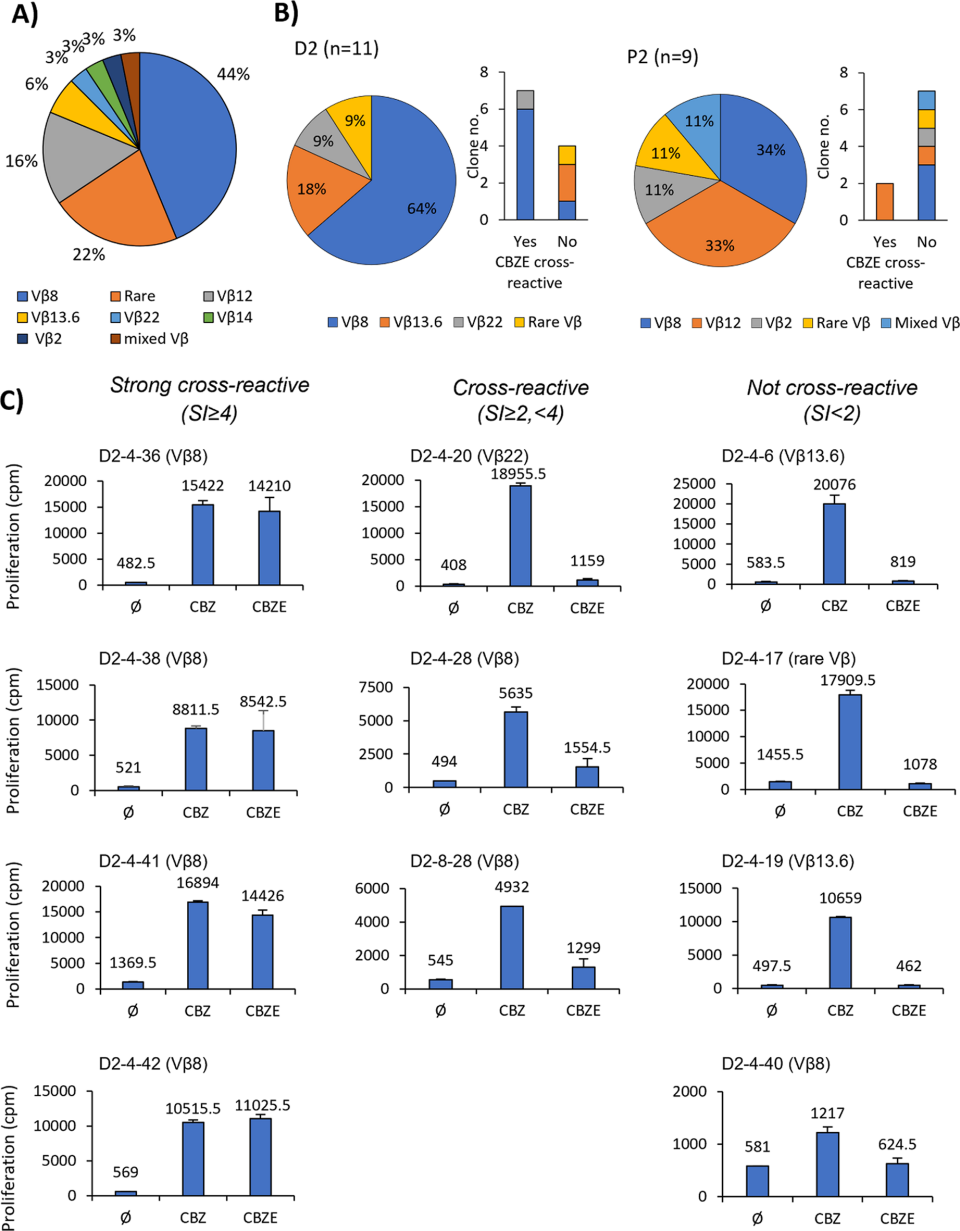
TCR Vβ phenotype of CBZ-specific T-cell clones with and without CBZE cross-reactivity. (A) Frequency of TCR Vβ phenotypes and number of T-cell clones with and without CBZE cross-reactivity that generated from D2 (*n* = 11) and patient P2 (*n* = 9). Only T-cell clones with known CBZE cross-reactive properties and TCR Vβ phenotype were shown. The TCR Vβ phenotyping was done by treating clones with fluorescently labeled antibodies and fluorescence was measured by flow cytometry. A minimum of 10^4^ cells were analyzed in each experiment. (B) TCR Vβ phenotype of clones from donor D2 with three different CBZE cross-reactive properties was shown. The CBZ and CBZE responsiveness was determined by proliferation analysis. T-cell clones (5 × 10^4^/well) were cultured for 48 h at 37 °C, 5% CO_2_ in duplicate with autologous EBV-transformed B-cells (1 × 10^4^/well) in the absence and presence of CBZ or CBZE (25 μg/mL). The proliferation activity was determined using the [^3^H]-thymidine incorporation assay. Data showed mean proliferation (cpm: counts per minute) and SEM. In the presence of CBZE, the stimulation index (SI = cpm with drug/ cpm without drug) of ≥4, between 2 and 4, and < 2 was assigned as strong cross-reactive, cross-reactive, and not cross-reactive, respectively. Ø: no drug control, CBZ: carbamazepine, and CBZE: carbamazepine-10,11-epoxide.

From a total of 1483 tested clones from P1, P2, D1, and D2, 73 clones were characterized as CBZ-specific T-cells (Table 1, Figure 1A). Four and twelve CBZ-specific T-cell clones were generated from the CBZ-naïve donor D1 and D2, respectively, while nineteen and thirty-eight were generated from CBZ hypersensitive patients P1 and P2, respectively. Out of these, 44/47 displayed the CD4^+^ phenotype. All tested clones proliferated in a dose-dependent manner and released IFN-γ in response to CBZ (Figure 1B, Supplementary Figure S1). The CBZ-responsive T-cells were found to be heterogeneous in the TCR Vβ phenotype (Figure 2A). Only TCR Vβ8 was found to be shared among individuals—D2 and P2 (Table 1). Among the CBZ-specific clones from D2, a different dose-responsiveness pattern and cross-reactivity were observed against the CBZ major metabolite, carbamazepine-10,11-epoxide (CBZE). The clones that displayed CBZE cross-reactivity expressed either Vβ8 or Vβ22, while the CBZ-specific clones with no cross-reactivity expressed Vβ8, Vβ13.6, and a rare TCR Vβ (Figure 2B). A strong cross-reactive response (stimulation index, SI, of equal or higher than 4) was observed only in clones with Vβ8 (Figure 2C).

**Table 1:** Number and Characteristics of Carbamazepine (CBZ)-Specific T-Cell Clones Generated^a^

donor/patient	high-risk HLA allele	responsiveness (specific clones/tested clones)^b^		CD phenotype (*n*)	TCR Vβ (*n*)
		CBZ	CBZE		
donor D1	A*31:01	4/241	3/3	CD4 (4)	not detected (4)
donor D2	B*15:02	12/154	7/11	CD4 (11), CD8 (1)	Vβ8 (7), Vβ13.6 (2), Vβ22 (1), not detected (1)
DRESS patient P1	A*31:01	19/140	0/2	CD4 (5), CD8 (1), not tested (13)	Vβ14 (1), not detected (3)
MPE patient P2	B*57:01	38/948	0/11	CD4 (24), mixed (1), not tested (13)	Vβ8 (7), Vβ12 (5), Vβ2 (1), mixed Vβ12 & Vβ7.1 (1)

a CBZE: carbamazepine-10,11-epoxide, DRESSs: drug reaction with eosinophilia and systemic symptoms, and MPE: maculopapular exanthema.

b Only T-cell clones with confirmed CBZ-responsiveness were tested for CBZE cross-reactivity.

### CD4^+^ T-Cells Responded to CBZ in an HLA-DR Restricted Manner

**Figure 3:**
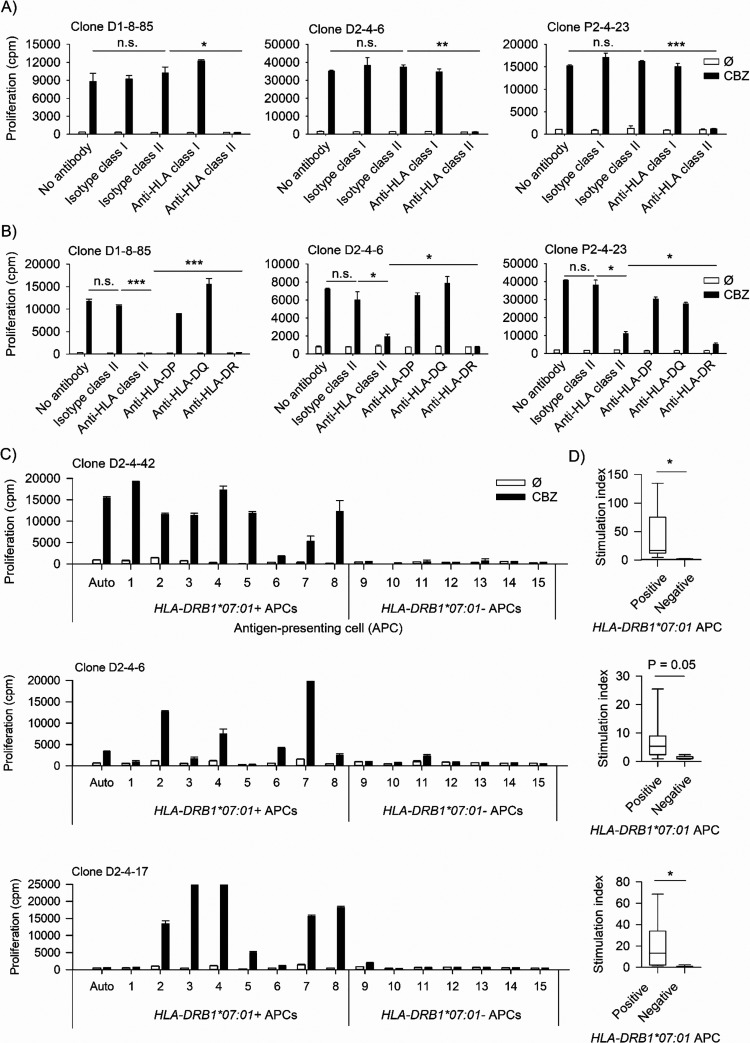
HLA blocking and HLA mismatch analyses identified HLA-DRB1*07:01 as a restricted marker. T-cell clones (5 × 10^4^/well) were cultured for 48 h at 37 °C, 5% CO_2_ in duplicate with EBV-transformed B-cells (1 × 10^4^/well) in the presence and absence of CBZ (25 μg/mL). The proliferation activity was determined using the [^3^H]-thymidine incorporation assay. Data showed mean proliferation (cpm: counts per minute) and SEM. (A, B) HLA blocking analyses were done by incubating the EBV-transformed B-cells with antibody for 1 h at 37 °C, 5% CO_2_, before using in the experiment. CBZ-mediated response of CD4^+^ T-cells from D1, D2, and P2 was blocked by anti-HLA class II (A) and anti-HLA-DR (B). (C) HLA mismatch analyses on a clone from D2 were done by incubating T-cell clones with a panel of EBV-transformed B-cells. The CBZ-mediated response was restricted to HLA-DRB1*07:01^+^ antigen-presenting cells (APCs). (D) The stimulation index (SI = cpm with drug/ cpm without drug) in the presence of HLA-DRB1*07:01^+^ APC was compared to the SI in the presence of HLA-DRB1*07:01^–^ APC using a *t* test. (Auto: autologous APC, Ø: no drug control, and *: *P* value < 0.05).

**Figure 4:**
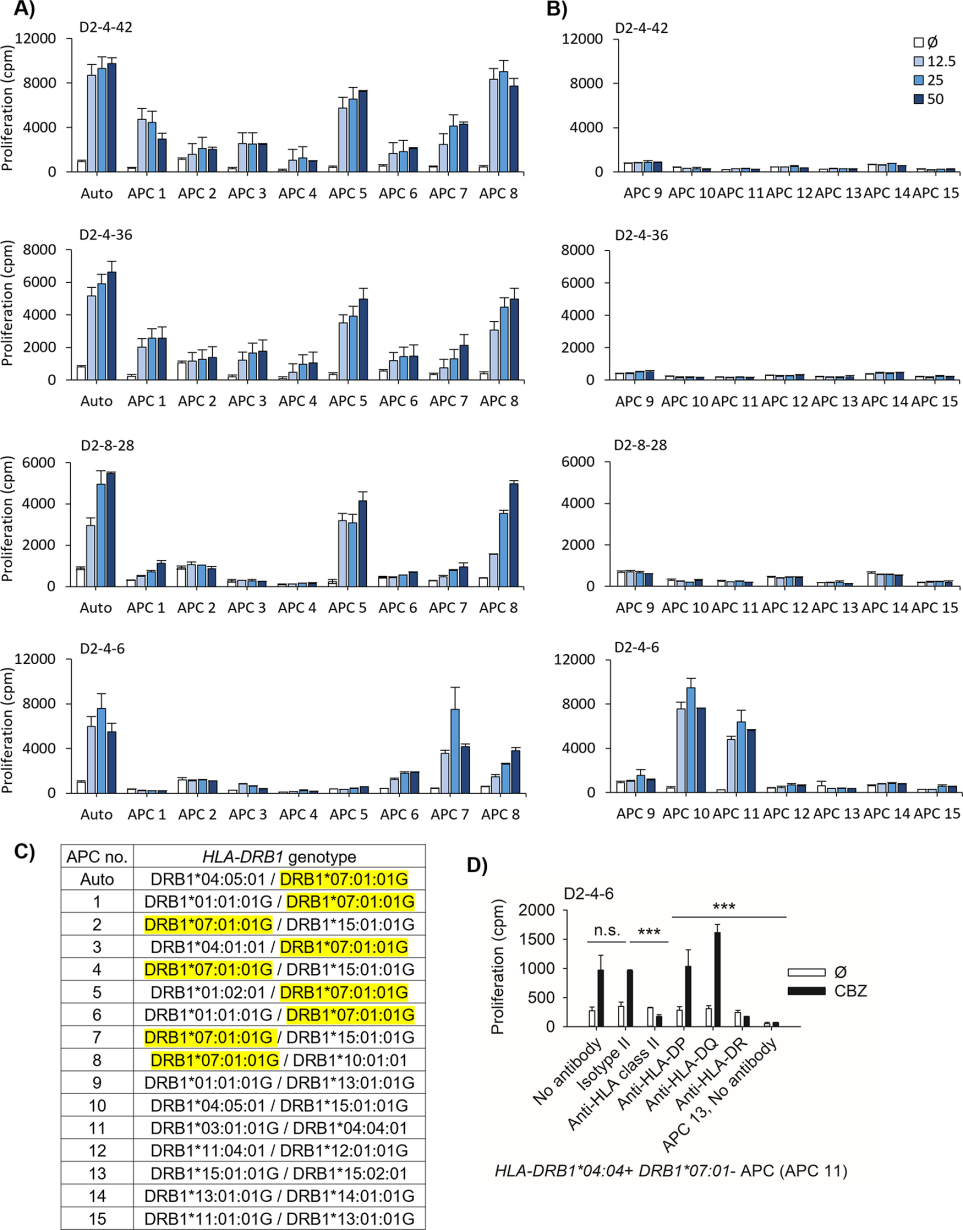
Dose-dependent CBZ-mediated response against HLA-DRB1*07:01^+^ and HLA-DRB1*07:01^–^ APCs of the T-cell clones from donor D2. T-cell clones (5 × 10^4^/well) were cultured for 48 h at 37 °C, 5% CO_2_ in duplicate with a panel of (A) HLA-DRB1*07:01^+^ EBV-transformed B-cells (1 × 10^4^/well) and a panel of (B) HLA-DRB1*07:01^–^ EBV-transformed B-cells in the presence and absence of graded concentration of CBZ (12.5–50 μg/mL). The proliferation activity was determined using the [^3^H]-thymidine incorporation assay. Data showed mean proliferation (cpm: counts per minute) and SEM. (C) *HLA-DRB1* genotype of each EBV-transformed B-cell was listed and the HLA-DRB1*07:01 allele was highlighted in yellow. (D) CBZ-mediated response of clone D2-4-6 in the presence of APC 11 was confirmed by the HLA blocking assay. EBV-transformed B-cells (1 × 10^4^/well) were incubated with the antibody for 1 h at 37 °C, 5% CO_2_, before incubating with the T-cell clone (5 × 10^4^/well) in the presence and absence of CBZ (25 μg/mL). The proliferation activity was determined using the [^3^H]-thymidine incorporation assay. Data showed mean proliferation (cpm) and SEM. Auto, autologous; Ø: no drug control; and APC: antigen-presenting cell (n.s., not significant; **, *P* value < 0.005; and ***, *P* value < 0.0005).

The CBZ-mediated response of CD4^+^ CBZ-specific T-cell clones was identified to be HLA-DR-restricted as both anti-HLA class II antibody and anti-HLA-DR antibody were able to inhibit antigen-specific proliferative responses (Figure 3A,B). In all tested clones, the proliferative T-cell response in the presence of isotype II antibody control was not different from those in control wells without antibodies (*P* > 0.05). The proliferative T-cell responses in the presence of anti-HLA class II and anti-HLA-DR were significantly lower than those observed in isotype control wells (*P* < 0.05). The CBZ-presenting HLA-DR molecule was further identified by HLA mismatch analyses. A panel of 16 APCs with different HLA genotypes was generated and utilized for this assay (Supplementary Table S2). Successful identification was possible in several clones whose mismatched response pattern was compatible with the HLA-DRB1*07:01 allele (Figure 3C). T-cell clones incubated in the presence of CBZ with HLA-DRB1*07:01^+^ APCs displayed a significantly higher SI than those co-incubated with HLA-DRB1*07:01^–^ APCs (*P* < 0.05, Figure 3D). The mismatched response was confirmed by dose–response analyses against mismatched APCs (Figure 4A–C). Apart from HLA-DRB1*07:01^+^ APCs, two additional HLA-DRB1*04^+^DRB1*07:01^–^ APCs (APC 10 and APC 11) also led to an HLA-DR-cross-reactive CBZ-mediated response in one clone (Figure 4B). Cross-reactivity against APC with HLA-DRB1*04:04 was confirmed by anti-HLA class II and anti-HLA-DR blocking analysis. The CBZ-mediated response was significantly lower in the presence of blocking antibodies (*P* < 0.05) (Figure 4D). The restricted HLA-DR markers of other clones could not be identified due to self-presenting activity, which allowed the T-cell clones to respond to CBZ regardless of APCs (Supplementary Figures S2 and S3).

### Absence of CBZ-Responsive T-Cells in Healthy Donors without HLA Class II Alleles, HLA-DRB1*07:01 and HLA-DRB1*04:04

As CBZ-specific CD4^+^ T-cell responses were restricted to HLA-DRB1*07:01 and HLA-DRB1*04:04, we next evaluated whether CBZ-responsive T-cells could be generated from donors expressing HLA class I markers, but not these HLA class II markers. PBMCs from two additional CBZ-naïve healthy donors were used in the experiments (donor D3 with HLA-A*31:01 and donor D4 with HLA-B*15:02). Both donors expressed no HLA-DR marker shared with the four initial donors/patients that led to the successful generation of CBZ-responsive T-cells with an HLA class II-restricted response (Supplementary Table S1). A total of 291 T-cell clones (150 for D3 and 141 for D4) were generated and tested for proliferative responses in the presence and absence of CBZ. No drug-responsive T-cells were identified from these two donors who carry no restricted HLA class II marker.

### HLA-DR Restricted CBZ-Mediated Response Is Processing Independent and Causes Granulysin Release

**Figure 5:**
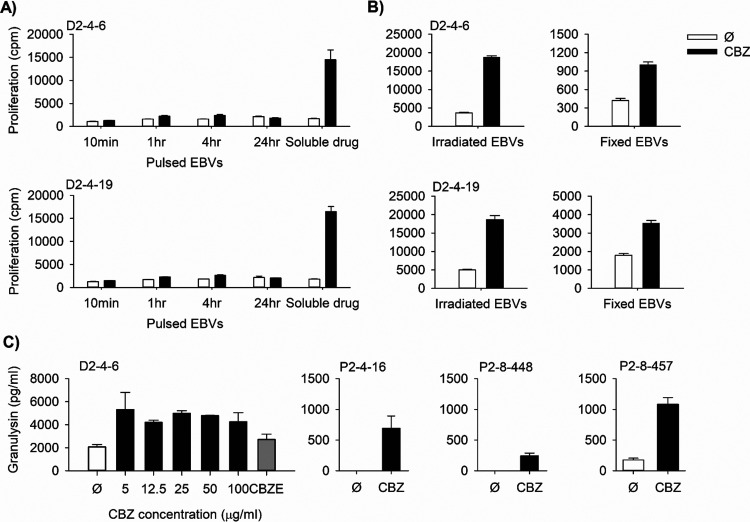
Characterization of the HLA-DR-restricted response mechanism. (A) Carbamazepine (CBZ) pulse analysis using EBV-transformed B-cells pulsed with CBZ for 10 min, 1 h, 4 h, and 24 h compared with EBV-transformed B-cells in soluble CBZ. A proliferative response was detected only in the presence of the soluble drug. (B) Glutaraldehyde fixation analysis comparing between CBZ-mediated response against unfixed irradiated EBV-transformed B-cells (left) and glutaraldehyde-fixed EBV-transformed B-cells (right) showed the presence of a proliferative response in both conditions. (C) Granulysin release analysis by ELISA on T-cell clones from D2 and P2 revealed CBZ-mediated granulysin release.

The molecular mechanism of how CBZ interacts with HLA-DR was studied using glutaraldehyde fixation of APC and CBZ APC pulsing analyses.^19,20^ CD4^+^ CBZ-specific T-cell clones were activated in the presence of irradiated non-fixed EBV-transformed B-cells with soluble CBZ but not against EBV-transformed B-cells pulsed with CBZ for various duration (Figure 5A). This suggested that the T-cells are activated with the parent drug or stable metabolite that does not form adducts. The T-cells were also activated with soluble CBZ in the presence of glutaraldehyde-fixed APCs which ruled out the possible involvement of intracellular processing of drug- (metabolite) protein adducts (Figure 5B). These findings indicate that the HLA-DR-restricted response is likely to occur via CBZ binding directly to surface HLA-DR molecules.

Granulysin-releasing activity in CD4^+^ CBZ-responsive T-cells was evaluated using human granulysin ELISA. Among nine tested CD4^+^ CBZ-specific T-cell clones, one clone from a healthy donor D2 and three clones from a hypersensitive patient P2 were found to release granulysin in response to CBZ (Figure 5C). Our results indicate that these HLA class II-restricted CD4^+^ T-cells may contribute to tissue injury through effector mechanisms comparable to CD8^+^ T-cells.

### Database Search for MHC Class II Associations and Its Haplotype Frequency

To substantiate the role indicated by our functional studies, the HLA Adverse Drug Reaction Database—part of the AFND—was reviewed for reported associations between CBZ hypersensitivity reactions and HLA class II markers (accessed on 21st April 2020).^21^ Five association studies were found; one study reporting only the allele frequency was excluded, while three studies from Han Chinese^15,22,23^ and one study from a North Indian population^24^ were included for recalculating statistical significance (Table 2). For CBZ-induced SJS–TEN, five HLA class II markers, including HLA-DRB1*07:01, showed a significant association in Han Chinese. HLA-DRB1*04:05 was the only allele with a negative correlation with CBZ-induced SJS–TEN. The association between HLA-DRB1*07:01 and CBZ-induced SJS–TEN was marginally significant in the North Indian population (*P* = 0.052, odds ratio = 7.25, 95% CI = 1.09–48.19). For MPE, a trend toward an association with HLA-DRB1*04:05 was found in Han Chinese (*P* = 0.064, odds ratio = 3.03, 95% CI = 1.07–8.58). However, no HLA class II marker was reported to be associated with DRESSs.

**Table 2:** Associations between Carbamazepine-Induced Hypersensitivity Reactions and HLA Class II Markers^a^

reactions	population	allele	case frequency (*n*)		*P*	odds ratio (95% CI)	ref
			patient case	tolerant control			
SJS–TEN	Han Chinese	DRB1*01:01	7.4% (4/54)	0.6% (1/176)	0.013	14 (1.53–128.10)	(23)
		DRB1*04:05	1.7% (1/60)	17.4% (25/144)	0.005	0.081 (0.01–0.61)	(15)
		DRB1*07:01	16.0% (8/20)	10.4% (13/125)	0.002	5.74 (1.98–16.63)	(22)
		DRB1*12:02	68.3% (41/60)	16.0% (23/144)	<0.001	11.35 (5.62–22.94)	(15)
			53.7% (29/54)	25.6% (45/176)	<0.001	3.38 (1.79–6.36)	(23)
		DQB1*03:01	16.0% (8/20)	20.8% (26/125)	0.11	2.54 (0.94–6.86)	(22)
		DQB1*03:03	50% (10/20)	17.6% (22/125)	0.037	2.80 (1.15–6.77)	(22)
	Indian	DRB1*07:01	60% (3/5)	17.1% (12/70)	0.052	7.25 (1.09–48.19)	(24)
DRESSs	Han Chinese	DRB1*04:05	7.7% (1/13)	17.4% (25/144)	0.611	0.40 (0.05–3.19)	(15)
		DRB1*12:02	23.1% (3/13)	16.0% (23/144)	0.787	1.58 (0.40–6.18)	(15)
MPE	Han Chinese	DRB1*04:05	38.9% (7/18)	17.4% (25/144)	0.064	3.03 (1.07–8.58)	(15)
		DRB1*12:02	11.1% (2/18)	16.0% (23/144)	0.848	0.66 (0.14–3.06)	(15)

a SJS–TEN: Stevens–Johnson syndrome and toxic epidermal necrolysis, DRESSs: drug reaction with eosinophilia and systemic symptoms, and MPE: maculopapular exanthema. Data accessed from the HLA Adverse Drug Reaction Database on April 21st, 2020. *P* value, odds ratio, and 95% confidence interval (95% CI) were recalculated.

As the HLA class I and HLA class II genetic markers commonly occur as a haplotype, a linkage between HLA class II markers (HLA-DRB1*01:01, DRB1*07:01, DRB1*12:02, and DQB1*03:03) and known high-risk HLA class I alleles (HLA-A*31:01, B*15:02, B*15:11, B*15:21, and B*57:01) was investigated. The haplotype frequencies were reviewed using the HLA haplotype frequency search within the AFND.^21^ The HLA-B*15:02-DRB1*12:02 haplotype was commonly found in various Asian populations, including Han Chinese, Malaysian, Filipino, and Vietnamese (Supplementary Table S3). Both HLA-DRB1*07:01 and DQB1*03:03 were frequently found linked with HLA-B*57:01 in South America, Europe, South Asia, and Southeast Asia. The haplotype frequency of HLA-B*57:01-DRB1*07:01 was reported to be above 5% in India, and the haplotype frequency of HLA-B*57:01-DQB1*03:03 was reported to be above 7% in Ireland, Sri Lanka, and Tunisia. Despite the relatively high haplotype frequency, evidence of HLA-B*57:01-DRB1*07:01-DQB1*03:03 haplotype association with CBZ hypersensitivity is limited. The review of the HLA Adverse Drug Reaction Database revealed only a single patient of Han Chinese descent carrying both HLA-B*57:01 and HLA-DRB1*07:01. Meanwhile, the HLA-A*31:01 allele was not commonly linked with *HLA-DRB1* or *HLA-DQB1* alleles. All reported haplotype frequencies comprising the HLA-A*31:01 allele were mostly lower than 1.5% frequency in all populations. Similarly, the haplotype comprising HLA-DRB1*01:01 was also reported in few populations with, mostly, less than 1% frequency (Supplementary Table S3).

## Discussion

Genetic association studies highlight a remarkable role for specific HLA class I alleles as genetic predictors of drug hypersensitivity reactions. Functional studies with T-cells from hypersensitive patients have shown that drugs bind with a degree of selectivity to the HLA proteins identified in the genetic association studies to stimulate a CD8^+^ T-cell response. Much less is known about the role of HLA class II in the pathogenesis of the reaction. In this study, HLA-DRB1*07:01 was identified as a restricted marker for the drug-mediated CD4^+^ T-cell response and a marker associated with CBZ hypersensitivity reactions. A variation in the HLA restriction pattern was also observed among CBZ-specific T-cell clones generated from both the healthy donors and CBZ hypersensitive patients (for instance, a clone with HLA-DRB1*07:01 restriction displayed HLA-DRB1*04:04 cross-reactivity). HLA class II was revealed to be important for CBZ-mediated CD4^+^ T-cell proliferative responses. The response against HLA-DRB1*04:04—noted in a clone derived from an HLA-B*15:02 expressing healthy donor in this study—was also previously reported in CD4^+^ T-cells from a hypersensitive patient with HLA-A*31:01.^11^ Collectively, there could be multiple HLA class II markers responsible for CD4^+^ T-cell activation in CBZ hypersensitivity reactions and this CD4^+^ T-cell-mediated mechanism could cooperate with the HLA class I marker to produce the different clinical manifestations seen in CBZ hypersensitivity.

CD4^+^ clones from patients with hypersensitivity and healthy donors positive for the HLA class I risk alleles were found to be heterogeneous, responding in an HLA class II-restricted manner and demonstrating CBZ-mediated granulysin-releasing activity. Previously, CD4^+^ CBZ-specific clones have been shown to release mediators, including IFN-γ, IL-4, and IL-5.^18,19^ These CD4^+^ clones were also shown to secrete granzyme B, perforin, and Fas ligand in response to CBZ, similar to CD8^+^ clones.^11^ The granulysin-releasing activity shown in this study indicates potent cytotoxic activity and an important role for CD4^+^ T-cells in the reaction pathogenesis. Granulysin release by CD4^+^ T-cells is not restricted to patients with drug hypersensitivity. For example, CD4^+^ T-cells have been reported to secrete granulysin in response to various intracellular pathogens, including Cryptococcus,^25^ tuberculosis,^26,27^ leprosy,^28^ and EBV.^29,30^

The TCR Vβ phenotype shows a polyclonal response in the generated CBZ-responsive T-cells. The Vβ phenotype of each clone varied among individuals, except Vβ8 which outnumbered the expression of other TCR Vβ phenotypes. This shared TCR Vβ8 usage between hypersensitive patients and healthy donors indicates a preferential use of a TCR that recognized CBZ in the context of the HLA molecule. Our previous study utilizing a similar approach found a different set of TCR Vβ in CD4^+^ and CD4^+^CD8^+^ clones, including Vβ5.1, Vβ13.5, and Vβ17.^18^ Notably, the TCR phenotypes of the CD4^+^ T-cells in our study overlapped with phenotypes reported in PBMCs and blister cells from CBZ hypersensitive patients in a different cohort.^31^ TCR Vβ8 (TRBV12–3, 12–4), Vβ13.6 (TRBV6–6), and Vβ14 (TRBV27) were shared in both this study and the previous study in which Vβ8 was similarly found to be the most common T-cell population. This Vβ8 phenotype, described as TRBV12–4 in the previous study, was reported in blister cells in 7/7 cases along with the preferential TCRβ CDR3, ASSLAGELF. Furthermore, the ASSLAGELF CDR3 clonotype was also found in PBMCs from CBZ hypersensitive patients with different ethnicities (Han Chinese, Thai, and European).^31^

Interestingly, Vβ13.6 was identified both in the CD4^+^ clone with granulysin-releasing activity in this study and in the blister cell population in a separate cohort.^31^ This shared phenotype was unexpected since the blister cells were described to be essentially CD8^+^ and were known to exhibit granulysin-mediated cytotoxicity. This finding indicates possible effector similarity between CD4^+^ and CD8^+^ T-cells crucial to the etiology of CBZ hypersensitivity, which may be an important contributor to the etiology of CBZ hypersensitivity. A recent single-cell sequencing study of CBZ-responsive CD8^+^ T-cells also showed a similar set of TCR Vβ clonality in CBZ-naïve healthy individuals and SJS–TEN patients who carry HLA-B*15:02.^32^ However, sharing similar TCR Vβ phenotype does not necessarily translate to a similar CDR3 repertoire. Only a small proportion of CD4^+^ and CD8^+^ TCR were reported to share similar TCRα and TCRβ when evaluated in high resolution.^33^ Additionally, the diversity of TCRs among individuals is still not fully understood, especially, in relation to different HLA genotypes. A further high-resolution investigation is required for a better understanding of clonality and functionality of the drug-specific T-cell response.

Our database review suggests that genetic predisposition to CBZ hypersensitivity is heterogeneous. The associations identified with HLA class I alleles are a reflection of the effect size of these HLA alleles.^34^ HLA class II alleles are likely to have a lower effect size and thus have not been identified in the restricted sample sizes so far studied in GWASs. This has also been seen in the Asian population where multiple HLA markers with CBZ-binding capacity overlap with each other, the most frequent HLA class I marker, HLA-B*15:02, reaching statistical significance ahead of less common markers.^5^ Uncommon HLA-B75 serotype markers have now been reported as associations with HLA-B*15:11 in Japanese and Korean,^3,4^HLA-B*15:21 in Thai,^7^ Indonesian,^6^ and Filipino^8^ populations. For HLA class II markers, the associations were reported in specific populations in whom the HLA-B*15:02 frequency was relatively low.^22,24^ Identification of other genetic factors relies on identifying larger numbers of affected patients, but given the rarity of the reactions, this may not always be possible. Consequently, the approach outlined in this study using functional analysis provides an alternative but complementary avenue for delineating the heterogeneity in genetic predisposition.

**Figure 6:**
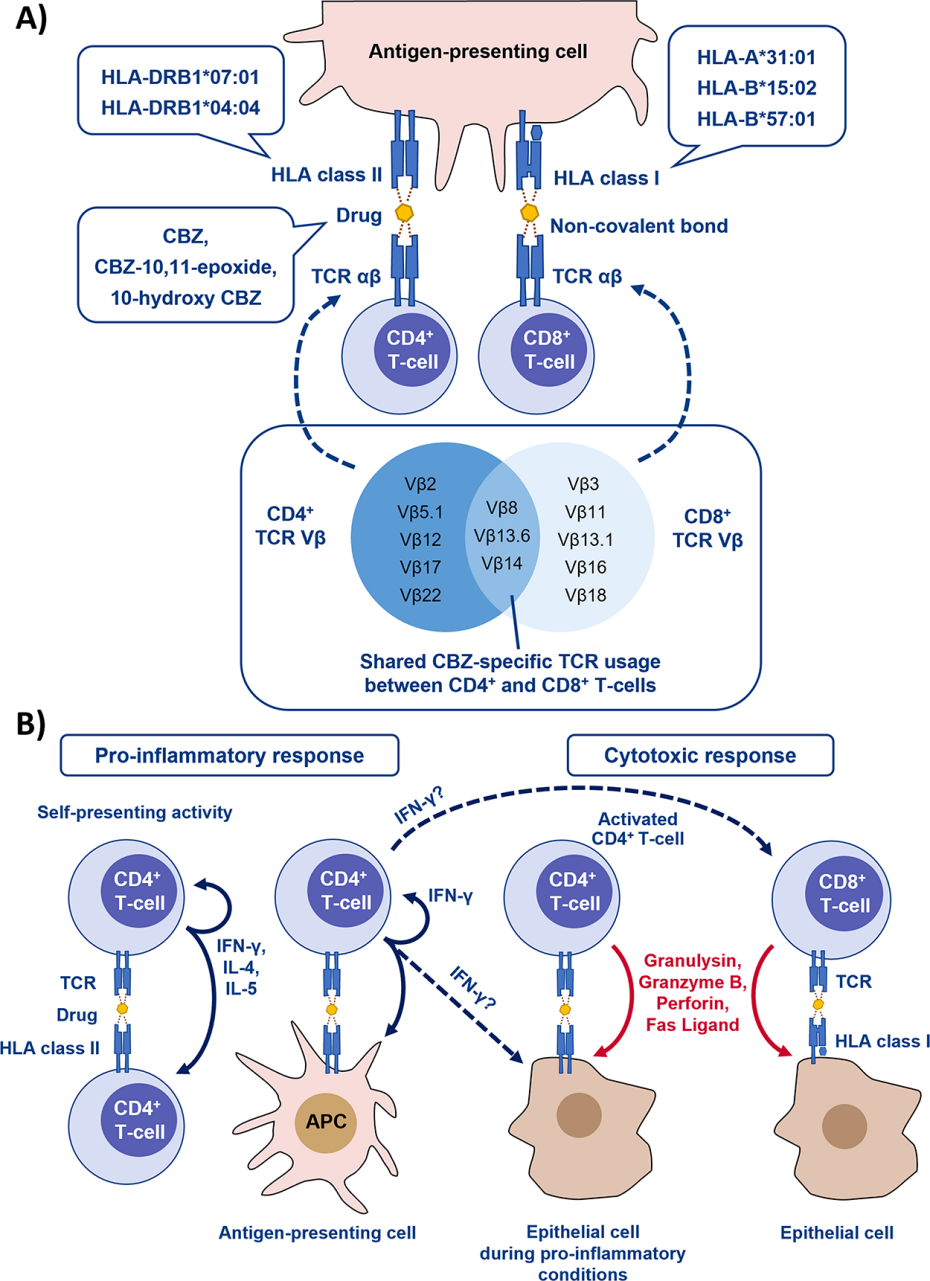
Proposed model of CD4^+^ and CD8^+^ T-cell function and related mechanisms in carbamazepine (CBZ) hypersensitivity. (A) Different combinations of drug/metabolite, HLA, and TCR molecules can be involved in the complex formation. Various HLA class I and II molecules can present CBZ (and its metabolites) to the TCR molecule without forming any covalent bond. Various TCRs can also recognize the presented drug while some TCR Vβs were shared between both CD4^+^ and CD8^+^ T-cell populations. (B) Function of the CD4^+^ T-cells and CD8^+^ T-cells activated by CBZ may consist of pro-inflammatory and cytotoxic responses. Dashed lines indicated unestablished roles of CD4^+^ T-cells in the pathogenesis of the reaction. Red lines indicated cytotoxic molecules secreted after immune activation.

The mechanism of cellular interaction by which these markers lead to the varied phenotypic manifestations of CBZ hypersensitivity is not understood. Based on available evidence herein and published elsewhere, we developed a model to summarize mechanistic findings exploring CBZ-specific T-cell responses (Figure 6). Initially, CBZ triggers either CD4^+^ or CD8^+^ T-cells by a formation of drug/peptide/HLA/TCR complex where different TCR, HLA, and drug molecules interact with each other in a complex manner. The drug-specific TCR on CBZ-specific T-cells could be either shared (e.g., Vβ8, Vβ13.6 which is shared between both CD4^+^ and CD8^+^ T-cells) or restricted (e.g., Vβ2 which is only found in CD4^+^ T-cells) (Figure 6A). Some drug-specific TCRs also have a unique property that allows the T-cell to have a cross-reactive response to CBZ metabolites, CBZE, and 10-hydroxy CBZ (Figure 6A). CD4^+^ T-cell responses are typically restricted to HLA class II, and according to our data, HLA-DRB1*07:01 and DRB1*04:04 are likely to be important. This HLA restriction only showed in T-cell clones that exhibited no self-presentation in mismatch analyses. Many of these CD4^+^ clones possessed the capacity to self-present in the absence of APCs (Figure 6B). Meanwhile, CD8^+^ T-cells respond in an HLA-A or HLA-B restricted fashion, in keeping with the known associations with HLA-A*31:01, HLA-B*15:02, and HLA-B*57:01 (Figure 6B). After stimulation, the CD8^+^ CBZ-specific T-cells encode a cytotoxic function by secreting granulysin, granzyme B, perforin, Fas ligand, and IFN-γ, along with a weak proliferative response. Conversely, the CD4^+^ CBZ-specific T-cells react with a strong proliferative response and possess both pro-inflammatory and/or cytotoxic effector pathways (Figure 6B). In reality, the mechanism is likely to be even more complex with some HLA alleles likely to protect against the development of CBZ hypersensitivity, but these are only likely to be identified through large-scale genetic studies.

A limitation of our study is that we were unable to recruit drug-tolerant controls expressing high-risk HLA class I alleles since CBZ prescription in HLA-B*15:02 carriers has been prohibited for almost a decade. However, previous studies recruiting patients before this date have compared T-cell responses in CBZ-tolerant and hypersensitive patients.^19^ CBZ-responsive T-cells were only detected in hypersensitive patients. Of particular importance, Wei et al.^12^ studied CBZ T-cell responses using PBMCs from tolerant and hypersensitive patients that expressed HLA-B*15:02. T-cell responses were detected in almost all hypersensitive patients, but not the tolerant patients expressing the risk allele. Future investigations are necessary in HLA-B*57:01^+^ SJS cases to explore cellular mechanisms (HLA class I-restricted CD8^+^ T-cells and HLA class II-restricted CD4^+^ T-cells) behind the genetic association. This is of particular importance as we identified HLA class II-restricted CD4^+^ T-cells that release granulysin in response to CBZ in a MPE patient that expressed HLA-B*57:01.

Although our study found T-cells were stimulated with CBZ and CBZ-10,11-epoxide directly with no need for antigen processing, this does not exclude the presence of CBZ metabolite hapten-responsive T-cells. For example, previous studies with sulfamethoxazole and dapsone, where synthetic protein-reactive metabolites are available, identified drug and hapten-responsive T-cells in the same hypersensitive patients.^35,36^ In ongoing studies, we are synthesizing CBZ metabolite-modified HLA class I and class II allele binding peptides to explore their immunogenicity. It is also possible that reactive metabolites of CBZ promote the release of damage-associated molecular patterns from tissue cells, providing co-stimulatory signals to APCs and promoting drug-specific T-cell responses. This has been described with the 2-hydroxyiminostilbene metabolite of CBZ^37^ and with drugs such as clozapine^38^ and gefitinib.^39^

In summary, we have identified an immune mechanism of the HLA class II-restricted CBZ-mediated response. The HLA class II molecules were able to present CBZ to CD4^+^ T-cells and could trigger T-cell responses in an HLA-DR-restricted manner. Our data suggest that in addition to HLA class I-restricted CD8^+^ T-cells, HLA class II-restricted CD4^+^ T-cells, with the ability to secrete pro-inflammatory cytokines and cytotoxic molecules, may also play a crucial role in the pathogenesis of CBZ hypersensitivity.

## Supporting Information Available

Demographic data, clinical information, and HLA genotypes of healthy donors and hypersensitive patients; the HLA genotype of antigen-presenting cells used in the HLA mismatched analysis; haplotype frequencies of HLA markers with reported association with carbamazepine hypersensitivity; IFN-γ releasing activity of the CD4^+^ carbamazepine-responsive T-cell clone tested by ELISpot; the CBZ-mediated response in the absence of antigen-presenting cells; and HLA mismatch analyses were unable to determine the restricted HLA allele in carbamazepine (CBZ)-responsive T-cell clones with self-presenting activity (PDF)

## Abbreviations

AFND: allele frequency net database
APC: antigen-presenting cell
CBZ: carbamazepine
CBZE: carbamazepine-10,11-epoxide
CD: cluster of differentiation
DRESS: drug reaction with eosinophilia and systemic symptom
EBV: Epstein–Barr virus
GWAS: genome-wide association study
HLA: human leukocyte antigen
IFN-γ: interferon-gamma
MPE: maculopapular exanthema
PBMC: peripheral blood mononuclear cell
SI: stimulation index
SJS–TEN: Stevens–Johnson syndrome and toxic epidermal necrolysis
TCR: T-cell receptor
