# Supplementary material for: Characterization of Drug-Specific CD4+ T-Cells Reveals Possible Roles of HLA Class II in the Pathogenesis of Carbamazepine Hypersensitivity Reactions
Source: Chem Res Toxicol. 2023 Apr 19;36(5):757–68. doi: 10.1021/acs.chemrestox.2c00414 (PMC10189794; doi:10.1021/acs.chemrestox.2c00414)
Supplement: Supplementary file 1 — tx2c00414_si_001.pdf [file tx2c00414_si_001.pdf]

Characterization of drug-specific CD4+ T-cells reveals possible roles of HLA class II in the pathogenesis of carbamazepine hypersensitivity reactions

Kanoot Jaruthamsophon (orcid: 0000-0003-1563-0024) [a,b], *Paul J. Thomson (orcid: 0000-0001-5431-0459)* [a], *Sean Hammond (orcid: 0000-0002-3352-3866)* [a,c], *Eunice Zhang (orcid: 0000-0003-1813-2207)* [a], *Ana Alfirevic (orcid: 0000-0002-2801-9817)* [a], *Chonlaphat Sukasem (orcid: 0000-0003-0033-5321)* [a,d,e], *Dean J. Naisbitt (orcid: 0000-0003-4107-7832)* [a,*], *Munir Pirmohamed (orcid: 0000-0002-7534-7266)* [a,*]

AUTHOR ADDRESS

[a]Centre for Drug Safety Science, Department of Pharmacology and Therapeutics, Institute of Systems, Molecular and Integrative Biology, University of Liverpool, Liverpool, L69 3GE, UK.

[b]Department of Pathology, Faculty of Medicine, Prince of Songkla University, Songkhla, 90110, Thailand.

[c]ApconiX, Alderley Park, Alderley Edge, Cheshire SK10 4TG, UK.

[d]Division of Pharmacogenomics and Personalized Medicine, Department of Pathology, Faculty of Medicine Ramathibodi Hospital, Mahidol University, Bangkok, 10400, Thailand.

[e]Laboratory for Pharmacogenomics, Somdech Phra Debaratana Medical Center (SDMC), Ramathibodi Hospital, Bangkok, 10400, Thailand.

*Co-corresponding author email: DJN: dnes@liverpool.ac.uk, MP: munirp@liverpool.ac.uk

Table of Contents

Supplementary Materials and Methods S3

1 Materials S3

2 Generation of Epstein-Barr Virus-transformed B-cells S3

3 T-cell cloning S3

4 Testing for CBZ specificity in T-cell clones S3

5 Phenotyping studies by flow cytometric analysis S4

6 Cytokine release assays S4

7 HLA blocking studies S4

8 Characterization of drug-binding mechanism S4

9 Database search S4

10 Supplementary references S5

Supplementary Table S1. Demographic data, clinical information, and HLA genotypes of healthy donors and hypersensitive patients. S6

Supplementary Table S2. HLA genotype of antigen-presenting cells used in HLA mismatched analysis. S7

Supplementary Table S3. Haplotype frequencies of HLA markers with reported association with carbamazepine hypersensitivity S8

Supplementary Figure S1. IFN-γ releasing activity of $CD4^+$ carbamazepine-responsive T-cell clone tested by ELISpot. S13

Supplementary Figure S2. CBZ-mediated response in the absence of antigen-presenting cells. S14

Supplementary Figure S3. HLA mismatch analyses were unable to determine restricted HLA allele in carbamazepine (CBZ)-responsive T-cell clones with self-presenting activity. S15

Supplementary Material

Supplementary Materials and Methods

1 Materials

R9 medium is comprised of RPMI 1640 (Sigma), 10% pooled human blood type AB serum (Innovative Research, USA), 25 mM HEPES buffer (Sigma), 2 mM L-glutamine (Sigma), 25 mg/ml transferrin (Sigma), 100 mg/ml streptomycin and 100 U/ml penicillin (Sigma). F1 media is comprised of RPMI 1640 (Sigma), 10% pooled FBS (Gibco), 25 mM HEPES buffer (Sigma), 2 mM L-glutamine (Sigma), 100 mg/ml streptomycin and 100 U/ml penicillin (Sigma).

2 Generation of Epstein-Barr Virus-transformed B-cells

To generate Epstein-Barr Virus (EBV)-transformed B-cells, 5×10^6 PBMCs were incubated with 5 ml supernatant of B95.8 cells (filtered with 0.2µm filter) at 5% CO_2 at 37°C overnight. The incubated cells were then centrifuged, re-suspended with 2 ml of F1 media and 1 µg/ml cyclosporin A, and plated into a 24-well plate. The EBV-transformed B-cells were fed with cyclosporin A-supplemented F1 media for 2 weeks, and then fed with F1 twice a week. Well-growing cells were transferred to a 25-ml flask as needed and used as an immortalised APC cell line. For use in downstream analyses, EBV-transformed B-cells were irradiated for 20 minutes to eliminate proliferative capacity before use.

3 T-cell cloning

T-cell clones (TCC) were generated by T-cell cloning methodologies as previously described. [1] Firstly, CBZ-incubated bulk culture was generated by incubating 1×10^6 peripheral blood mononuclear cells (PBMC) with CBZ (25 and 50 µg/ml), and fed with IL-2 supplemented R9 medium on day 6 and 9. On day 14, CBZ- bulks were separated into CD4 and CD8 cells by anti-human CD8 magnetic beads (Miltenyi Biotec) via positive selection, and isolated as monoclonal populations through limiting dilution in 96-well. TCCs were expanded in 96-well plates via repetitive mitogen driven expansion (PHA) in the presence of irradiated allogeneic PBMCs from healthy donors every 14 days, punctuated by maintenance feeding with IL-2 supplemented R9 medium every 2 days. Well-growing clones were picked from the serial dilution, further expanded across 4 wells for a further 14 days.

4 Testing for CBZ specificity in T-cell clones

In order to assess compound responsiveness of TCC, clones exhibiting adequate expansion were drawn and split into 4 wells in an additional testing plate (5×10^4 cells per well). TCCs were incubated with 1×10^4 irradiated APC per well in R9 medium with and without the drug in duplicate in 96-well plate. The plate was incubated at 37°C, 5% CO_2 for 48 hours. 0.5 µCi [^{3}H]-thymidine was then added to each well, and incubated for an additional 16 hours. Proliferation activity was assessed through incorporated tritiated thymidine measured by a Beta scintillation counter (Perkin Elmer) as a function of counts per min (cpm). CBZ-specific proliferative response was defined as a stimulation index (SI; equal to cpm with drug divided by cpm without drug) of equal or greater than 1.5. SI ≥ 2 and ≥ 4 was interpreted as good and strong response respectively.

5 Phenotyping studies by flow cytometric analysis

Characterization of CD4/CD8 phenotype was performed using anti-CD4-FITC antibody (Miltenyi Biotec) and anti-CD8-antibody (Miltenyi Biotec) according to manufacturer instructions. A BD FACS Canto II flow cytometer (BD biosciences) was used for the fluorescence analysis. TCR Vβ phenotype was tested by using a panel of antibody cocktails, IOTest Beta Mark TCR Vβ Repertoire kit (Beckman Coulter). TCCs not categorised by the panel of antibody cocktails were classified as possessing rare TCR Vβ phenotypes.

6 Cytokine release assays

The IFN-γ ELISpot (Abcam) analysis was done in duplicate by incubating TCC (5×10^4) with irradiated autologous APC (1×10^4) for 48 hours (37°C, 5% CO_2) before read-out analysis, conducted according to manufacturer's instructions. Granulysin released by CBZ-TCCs was evaluated by ELISA (Abcam). The 100 µl supernatant was collected for the ELISA testing after the 48-hour incubation of proliferation assay and replaced with R9 media before adding [^{3}H]-thymidine. A total of 20 µl supernatant was used for the ELISA according to manufacturer instructions.

7 HLA blocking studies

HLA class II blocking was performed using anti-HLA-DP, anti-HLA-DQ, and anti-HLA-DR (Abcam). The tested APCs and TCCs were pre-incubated with 3 µl antibody for 1 hour before addition of CBZ or media control for proliferation analysis. HLA blocking studies were performed alongside parallel cultures treated with class I and II isotype antibodies serving as internal controls for anti-HLA class I and anti-HLA class II antibodies (Abcam) respectively.

8 Characterization of drug-binding mechanism

Carbamazepine APC pulsing analysis was used to determine whether the drug formed covalent bonds and whether the drug requires antigen processing for presentation to T-cells. [2] The pulsing process was done by incubating APCs with CBZ for 10 minutes, 1 hour, 4 hours, and 24 hours before extensive washing to remove unbound soluble drug. The pulsed APCs were then co-incubated with TCCs and proliferation was analyzed as previously described, TCCs co-incubated with APCs in the presence of soluble CBZ were used as a comparator. Glutaraldehyde fixation analysis was used for determining possible role of intracellular metabolism/processing in the formation of drug antigen. Glutaraldehyde (0.05%; Sigma Aldrich) fixation abolishes the antigen processing capabilities of APCs. Autologous APCs were fixed with glutaraldehyde for 30 seconds, quenched by glycine addition, and washed extensively. The glutaraldehyde fixed APCs were then used for the proliferation analysis. TCCs and CBZ incubated in the presence of unfixed APCs were used as positive control of glutaraldehyde fixation analysis.

9 Database search

Reported associations between HLA class II markers and CBZ hypersensitivity were searched using the HLA Adverse Drug Reaction Database (http://allelefrequencies.net/hla-adr/adr_query.asp) within the publicly available Allele Frequency Net Database (AFND). [3] All HLA class II markers reported to be associated with CBZ-induced adverse reactions were explored in all ethnicities. Full-text articles of all reported associations were reviewed. Association studies which describe only allele frequency were excluded from the analysis. The haplotype frequencies of known high-risk HLA class

I marker linked with HLA class II markers were reviewed as a 4-digit allele using the Haplotype Frequency Search tool (http://allelefrequencies.net/hla6003a.asp) within AFND. The frequencies of interested haplotype linked with any alleles in other loci were checked and re-calculated. All redundant haplotype frequencies reported from the same reference were excluded.

10 Supplementary references

1. Naisbitt DJ, Britschgi M, Wong G, et al. Hypersensitivity reactions to carbamazepine: characterization of the specificity, phenotype, and cytokine profile of drug-specific T cell clones. *Mol Pharmacol*. 2003;63(3):732-741.

2. Pichler WJ, Naisbitt DJ, Park BK. Immune pathomechanism of drug hypersensitivity reactions. *J Allergy Clin Immunol*. 2011;127(3 Suppl):S74-81.

3. Gonzalez-Galarza FF, McCabe A, Santos EJMD, et al. Allele frequency net database (AFND) 2020 update: gold-standard data classification, open access genotype data and new query tools. *Nucleic Acids Res*. 2020;48(D1):D783-D788.

Supplementary Table S1. Demographic data, clinical information, and HLA genotypes of healthy donors and hypersensitive patients. Underlined allele indicate HLA class I allele known to be associated with carbamazepine hypersensitivity.

ID	Clinical presentation	Ethnic origin	Time to reaction	Reaction onset (year)	Lymphocyte transformation test (Drug, SI)	HLA class I genotype	HLA class II genotype
Donor D1	Not applicable	White	Not applicable	Not applicable	Negative	*A*01:01* / *A*31:01*<br>*B*07:02* / *B*52:01*<br>*C*07:02* / *C*12:02*	*DRB1*15:01* / *DRB1*15:02*<br>*DQB1*06:01* / *DQB1*06:02*<br>*DQA1*01:03* / *DQA1*01:02*
Donor D2	Not applicable	Mixed (Mauritian and White)	Not applicable	Not applicable	Negative	*A*11:01* / *A*24:07*<br>*B*13:02* / *B*15:02*<br>*C*04:01* / *C*08:01*	*DRB1*04:05* / *DRB1*07:01*<br>*DQB1*02:01* / *DQB1*04:02*<br>*DQA1*02:01* / *DQA1*03:01*
Donor D3	Not applicable	Bangladeshi	Not applicable	Not applicable	Negative	*A*11:01* / *A*31:01*<br>*B*40:02* / *B*40:01*<br>*C*03:03* / *C*03:04*	*DRB1*13:01* / *DRB1*14:01*<br>*DQB1*05:03* / *DQB1*06:03*<br>*DQA1*01:03* / *DQA1*01:01*
Donor D4	Not applicable	Bangladeshi	Not applicable	Not applicable	Negative	*A*24:02* / *A*24:02*<br>*B*15:02* / *B*35:03*<br>*C*08:01* / *C*12:03*	*DRB1*11:01* / *DRB1*13:01*<br>*DQB1*03:01* / *DQB1*06:03*<br>*DQA1*01:03* / *DQA1*05:01*
HSS Patient P1	Generalized maculopapular rash with fever, eosinophilia & lymphocytosis	White	21 days	1989	CBZ, 3.4<br>CBZE, 2.4	*A*11:01* / *A*31:01*<br>*B*27:05* / *B*40:01*<br>*C*01:02* / *C*03:04*	*DRB1*03:01* / *DRB1*04:04*<br>*DQB1*02:01* / *DQB1*03:02*<br>*DQA1*03:01* / *DQA1*05:01*
MPE Patient P2	Generalised erythematous rash	White	6 days	1989	CBZ, 6.3<br>CBZE, 3.1	*A*01:01* / *A*24:02*<br>*B*50:01* / *B*57:01*<br>*C*06:02* / *C*06:02*	*DRB1*03:01* / *DRB1*07:01*<br>*DQB1*02:01* / *DQB1*03:03*<br>*DQA1*02:01* / *DQA1*05:01*

SI: stimulation index; HSS: hypersensitivity syndrome; MPE: maculopapular exanthema

Supplementary Table S2. HLA genotype of antigen-presenting cells used in HLA mismatched analysis.

**No.**	**EBV ID.**	**HLA class I**						**HLA class II**					
		HLA-A		***HLA-B***		***HLA-C***		***HLA-DRB1***		***HLA-DQB1***		***HLA-DQA1***	
Auto†	854	*A*11:01:01G*	*A*24:07*	*B*13:02:01G*	*B*15:02:01G*	*C*04:01:01G*	*C*08:01:01G*	*DRB1*04:05:01*	*DRB1*07:01:01G*	*DQB1*02:01:01G*	*DQB1*04:02:01G*	*DQA1*02:01*	*DQA1*03:01:01G*
1	560	*A*01:01:01G*	*A*02:01:01G*	*B*45:01:01G*	*B*51:01:01G*	*C*01:02:01G*	*C*06:02:01G*	*DRB1*01:01:01G*	*DRB1*07:01:01G*	*DQB1*02:01:01G*	*DQB1*05:01:01G*	*DQA1*01:01:01G*	*DQA1*02:01*
2	870	*A*02:01:01G*	*A*68:01:02G*	*B*07:02:01G*	*B*45:01:01G*	*C*06:02:01G*	*C*07:02:01G*	*DRB1*07:01:01G*	*DRB1*15:01:01G*	*DQB1*02:01:01G*	*DQB1*06:02:01G*	*DQA1*01:02:01G*	*DQA1*02:01*
3	853	*A*24:02:01G*	*A*29:02:01G*	*B*35:03:01G*	*B*45:01:01G*	*C*01:02:01G*	*C*06:02:01G*	*DRB1*04:01:01*	*DRB1*07:01:01G*	*DQB1*03:01:01G*	*DQB1*03:03:02G*	*DQA1*02:01*	*DQA1*03:01:01G*
4	465	*A*01:01:01G*	*A*24:02:01G*	*B*07:02:01G*	*B*44:03:01G*	*C*03:03:01G*	*C*07:01:01G*	*DRB1*07:01:01G*	*DRB1*15:01:01G*	*DQB1*02:01:01G*	*DQB1*06:02:01G*	*DQA1*01:02:01G*	*DQA1*02:01*
5	751	*A*01:01:01G*	*A*33:01:01*	*B*14:02:01*	*B*44:05:01*	*C*02:02:02G*	*C*08:02:01G*	*DRB1*01:02:01*	*DRB1*07:01:01G*	*DQB1*03:03:02G*	*DQB1*05:01:01G*	*DQA1*01:01:01G*	*DQA1*02:01*
6	802	*A*03:01:01G*	*A*33:03:01G*	*B*27:05:02G*	*B*44:03:02*	*C*02:02:02G*	*C*07:01:01G*	*DRB1*01:01:01G*	*DRB1*07:01:01G*	*DQB1*02:01:01G*	*DQB1*05:01:01G*	*DQA1*01:01:01G*	*DQA1*02:01*
7	976	*A*02:01:01G*	*A*03:01:01G*	*B*44:03:01G*	*B*44:02:01G*	*C*05:01:01G*	*C*16:01:01G*	*DRB1*07:01:01G*	*DRB1*15:01:01G*	*DQB1*02:01:01G*	*DQB1*06:02:01G*	*DQA1*01:02:01G*	*DQA1*02:01*
8	411	*A*02:01:01G*	*A*31:01:02G*	*B*15:01:01G*	*B*44:02:01G*	*C*03:03:01G*	*C*05:01:01G*	*DRB1*07:01:01G*	*DRB1*10:01:01*	*DQB1*02:01:01G*	*DQB1*05:01:01G*	*DQA1*01:01:01G*	*DQA1*02:01*
9	672	*A*03:01:01G*	*A*29:02:01G*	*B*45:01:01G*	*B*51:01:01G*	*C*02:02:02G*	*C*06:02:01G*	*DRB1*01:01:01G*	*DRB1*13:01:01G*	*DQB1*05:01:01G*	*DQB1*06:03:01G*	*DQA1*01:03:01G*	*DQA1*01:01:01G*
10	790	*A*02:60:01*	*A*03:01:01G*	*B*07:02:01G*	*B*45:01:01G*	*C*06:02:01G*	*C*07:02:01G*	*DRB1*04:05:01*	*DRB1*15:01:01G*	*DQB1*03:02:01G*	*DQB1*06:02:01G*	*DQA1*01:02:01G*	*DQA1*03:01:01G*
11	816	*A*02:01:01G*	*A*29:02:01G*	*B*40:01:01G*	*B*45:01:01G*	*C*03:04:01G*	*C*06:02:01G*	*DRB1*03:01:01G*	*DRB1*04:04:01*	*DQB1*02:01:01G*	*DQB1*03:02:08*	*DQA1*03:01:01G*	*DQA1*05:01:01G*
12	867	*A*01:01:01G*	*A*02:01:01G*	*B*13:02:01G*	*B*44:02:01G*	*C*05:01:01G*	*C*06:02:01G*	*DRB1*11:04:01*	*DRB1*12:01:01G*	*DQB1*03:01:01G*	*DQB1*06:02:01G*	*DQA1*01:02:01G*	*DQA1*05:01:01G*
13	422	*A*01:01:01G*	*A*31:01:02G*	*B*07:02:01G*	*B*52:01:01G*	*C*07:02:01G*	*C*12:02:01G*	*DRB1*15:01:01G*	*DRB1*15:02:01*	*DQB1*06:01:01G*	*DQB1*06:02:01G*	*DQA1*01:03:01G*	*DQA1*01:02:01G*
14	498	*A*11:01:01G*	*A*31:01:02G*	*B*40:02:01G*	*B*40:01:01G*	*C*03:03:01G*	*C*03:04:01G*	*DRB1*13:01:01G*	*DRB1*14:01:01G*	*DQB1*05:03:01G*	*DQB1*06:03:01G*	*DQA1*01:03:01G*	*DQA1*01:01:01G*
15	708	*A*24:02:01G*	*A*24:02:01G*	*B*15:02:01G*	*B*35:03:01G*	*C*08:01:01G*	*C*12:03:01G*	*DRB1*11:01:01G*	*DRB1*13:01:01G*	*DQB1*03:01:01G*	*DQB1*06:03:01G*	*DQA1*01:03:01G*	*DQA1*05:01:01G*

†Auto: autologous control for T-cell clones from Donor D2

Supplementary Table S3. Haplotype frequencies of HLA markers with reported association with carbamazepine hypersensitivity. Only populations with haplotype frequency of more than 0.10% were shown. Populations with more than 5% haplotype frequency were highlighted in **bold**. Data were accessed on 21st April 2020.

HLA class I	HLA class II	Populations	Frequency (%)
*A*31:01*	*DRB1*01:01*	Brazil	0.35
		Columbia	0.31
		Costa Rica	0.45
		Germany - ethnic minorities	<0.10-0.55
		India	0.34-1.12
		Italy	0.44
		Japan	0.29-0.36
		Malaysia	<0.10-0.18
		Mexico	0.15-0.69
		Poland	0.25-0.27
		Portugal	1.10
		Russia	0.23
		Spain	0.14
		Sri Lanka	0.70
		USA	<0.10-0.52
	*DRB1*07:01*	Brazil	0.67
		Chile	0.77
		Colombia	0.14
		England	0.20
		Gaza	1.28
		Germany - ethnic minorities	<0.10-0.31
		India	0.31-0.70
		Israel	<0.10-0.25
		Italy	0.52
		Malaysia	0.37
		Mexico	0.30-0.60
		New Zealand	0.48
		Nicaragua	0.32
		Poland	0.23
		Portugal	1.00
		Russia	0.17
		Spain	0.23-0.38
		Sri Lanka	0.21
		USA	<0.10-0.14
	*DRB1*12:02*	China	1.04

HLA class I	HLA class II	Populations	Frequency (%)
*A*31:01*	*DRB1*12:02*	Germany - ethnic minorities	<0.10-0.23
		Hong Kong	0.15-0.21
	*DQB1*03:03*	Brazil	1.33
		Chile	0.77
		England	0.20
		India	0.16-0.28
		Italy	0.28
		Japan	1.53
		Malaysia	0.37
		Mexico	0.10-0.61
		Peru	1.41
		Poland	0.12
		Russia	0.17
		USA	<0.10-0.31
*B*15:02*	*DRB1*01:01*	Less than 0.10%	
	*DRB1*07:01*	India	<0.10-0.29
		Malaysia	0.18-1.72
		United Arab Emirates	0.96
		Vietnam	0.99
	*DRB1*12:02*	**China**	**2.30-34.32**
		Germany - ethnic minorities	<0.10 - 2.19
		**Hong Kong**	**5.03-5.55**
		India	0.14 - 0.93
		**Malaysia**	**0.37-12.96**
		Mexico	0.43
		**Philippines**	**21.00**
		Sri Lanka	1.47
		**Taiwan**	**1.00-9.00**
		**USA**	**<0.10-7.82**
		**Vietnam**	**7.92-8.70**
	*DQB1*03:03*	India	<0.10-0.29
		Malaysia	0.11-0.37
		Mexico	0.27-0.61
		Sri Lanka	0.14
		USA	0.25
*B*15:11*	*DRB1*01:01*	Less than 0.10%	
	*DRB1*07:01*	Less than 0.10%	
	*DRB1*12:02*	Vietnam	0.50

HLA class I	HLA class II	Populations	Frequency (%)
*B*15:11*	*DQB1*03:03*	India	0.14
		Japan	0.5
		Malaysia	0.11-0.26
		USA	0.28
*B*15:21*	*DRB1*01:01*	Less than 0.10%	
	*DRB1*07:01*	Malaysia	0.11
	*DRB1*12:02*	Malaysia	0.26
		USA	0.20
	*DQB1*03:03*	USA	1.12
*B*57:01*	*DRB1*01:01*	Brazil	1.04
		Colombia	<0.10-1.06
		Costa Rica	0.23
		England	0.20
		Germany - ethnic minorities	<0.10-0.38
		India	<0.10-0.14
		Italy	1.57
		Poland	0.10
		Portugal	1.00-1.30
		Spain	<0.10-0.23
	*DRB1*07:01*	Brazil	3.33
		Cape Verde	1.60
		Chile	2.31
		Colombia	0.72
		Costa Rica	0.45
		Croatia	0.32
		England	2.90
		Germany - ethnic minorities	0.58-2.66
		Hong Kong	0.16-0.21
		**India**	**2.87-6.18**
		Iran	0.78
		Northern Ireland	1.80
		**Ireland**	**11.70**
		Israel	<0.10-1.58
		**Italy**	**5.09**
		Malaysia	0.26-4.98
		Mexico	0.45-1.07
		New Zealand	0.48
		Nicaragua	0.22
		Poland	2.00-2.34

HLA class I	HLA class II	Populations	Frequency (%)
*B*57:01*	*DRB1*07:01*	Portugal	1.00-1.80
		Russia	0.48-2.11
		South Africa	0.60-2.22
		Spain	1.54-1.86
		**Sri Lanka**	**7.07**
		**Tunisia**	**12.00**
		USA	<0.10-3.08
		Vietnam	0.50-2.10
	*DQB1*03:03*	Brazil	3.33
		Chile	2.31
		Colombia	0.68
		Costa Rica	0.45
		England	2.90
		Germany - ethnic minorities	0.55-1.71
		**India**	**2.87-5.66**
		Iran	0.78
		**Ireland**	**11.70**
		Italy	2.17
		Malaysia	0.47-4.61
		Mexico	0.15-1.07
		Nicaragua	0.22
		Poland	1.00-2.28
		Portugal	1.75
		Russia	1.94
		South Africa	0.60
		Spain	1.16-1.26
		**Sri Lanka**	**7.07**
		**Tunisia**	**9.00**
		United Arab Emirates	0.96
		USA	<0.10-2.18
		Vietnam	0.50-2.10
	*DRB1*07:01-DQB1*03:03*	Brazil	3.33
		Chile	2.31
		Colombia	0.68
		Costa Rica	0.45
		England	2.90
		Germany - ethnic minorities	0.55-1.62
		**India**	**2.87-5.65**

HLA class I	HLA class II	Populations	Frequency (%)
*B*57:01*	*DRB1*07:01-DQB1*03:03*	Iran	0.78
		**Ireland**	**11.70**
		Italy	1.89
		Malaysia	0.42-3.87
		Mexico	0.15-1.07
		Nicaragua	0.22
		Poland	1.00-2.24
		Portugal	1.32
		Russia	1.94
		South Africa	0.60
		Spain	1.16-1.26
		**Sri Lanka**	**7.00**
		**Tunisia**	**9.00**
		USA	<0.10-2.18
		Vietnam	0.50-2.10
	*DRB1*12:02*	Malaysia	<0.10-3.45

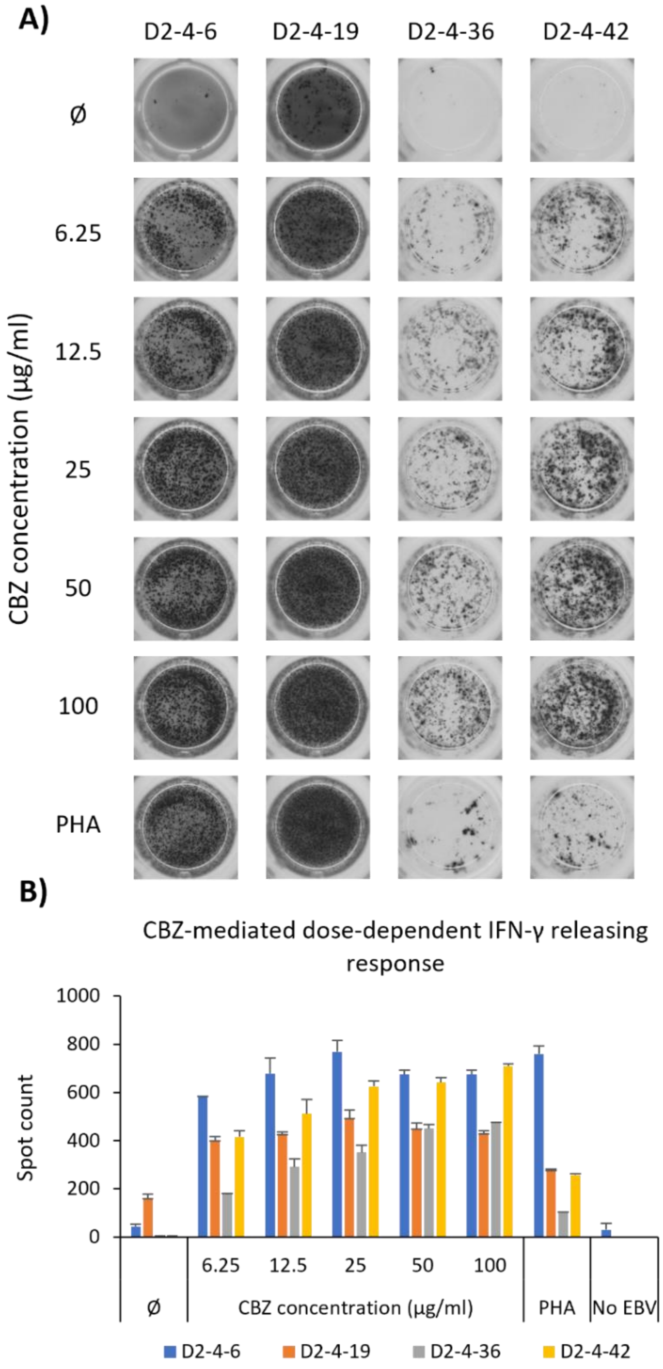

Supplementary Figure S1. IFN-γ releasing activity of CD4$^+$ carbamazepine-responsive T-cell clone tested by ELISpot. T-cell clones (5×10^4/well) were cultured for 48 hours at 37ºC, 5% CO_2 in duplicate with autologous EBV-transformed B-cells (1×10^4/well) in the presence and absence of graded concentration of CBZ (6.25-100µg/mL). The cytokine-secreting activity was determined using ELISpot (Abcam) as manufacturer protocol. The developed spots were captured (A) and counted (B) by ELISpot reader system. CBZ: carbamazepine, IFN-γ: interferon-gamma, PHA: phytohemagglutinin.

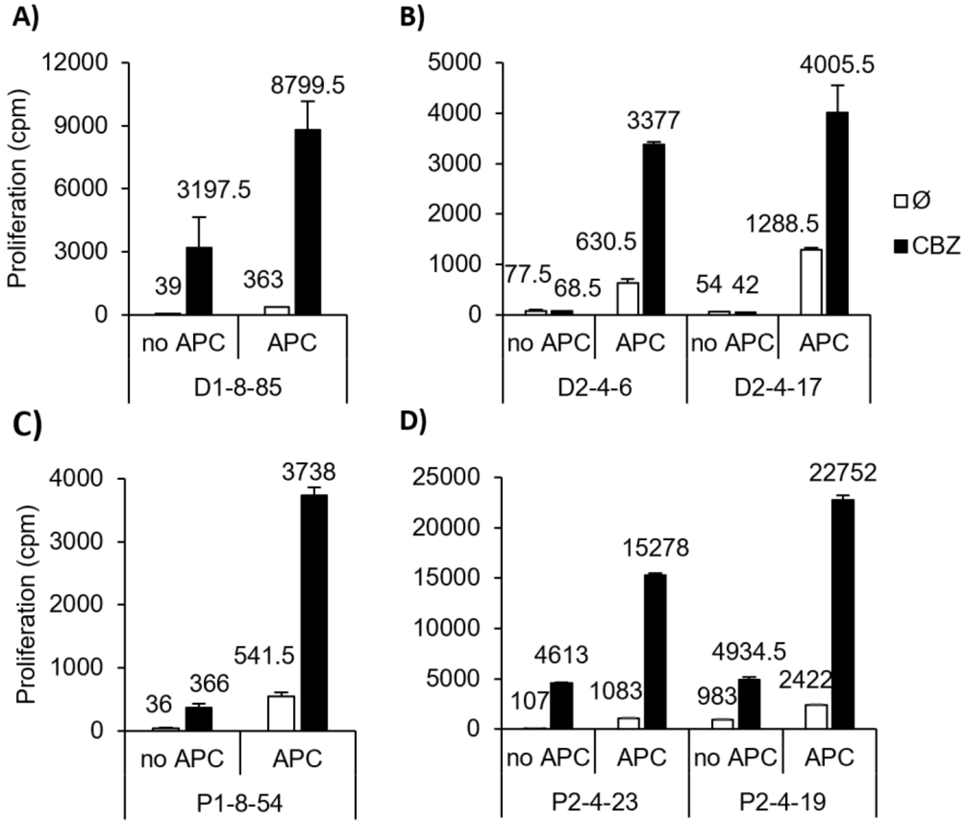

Supplementary Figure S2. CBZ-mediated response in the absence of antigen-presenting cells. T-cell clones (5×10^4/well) from donor D1 (A), donor D2 (B), patient P1 (C), and patient P2 (D) were cultured for 48 hours at 37ºC, 5% CO_2 in duplicate in the presence and absence of CBZ (25µg/mL), in the presence and absence of 1×10^4/well of autologous EBV-transformed B-cell (as an antigen-presenting cell, APC). The proliferation activity was determined using [^{3}H]-thymidine incorporation assay. Data showed mean proliferation (cpm: counts per minute) and SEM.

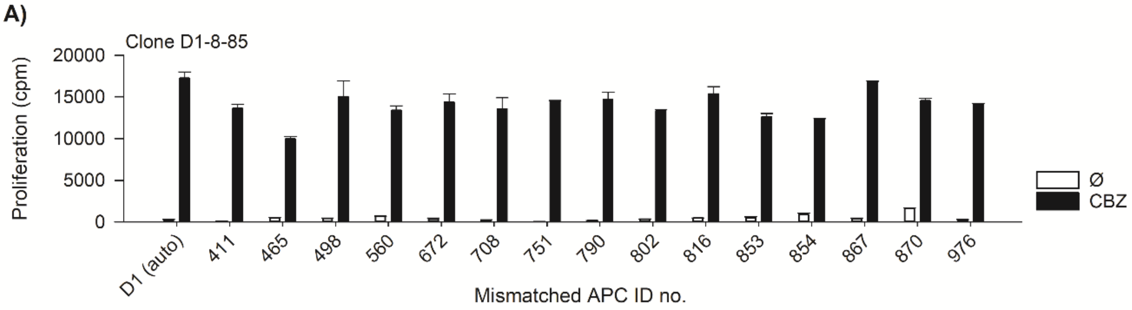

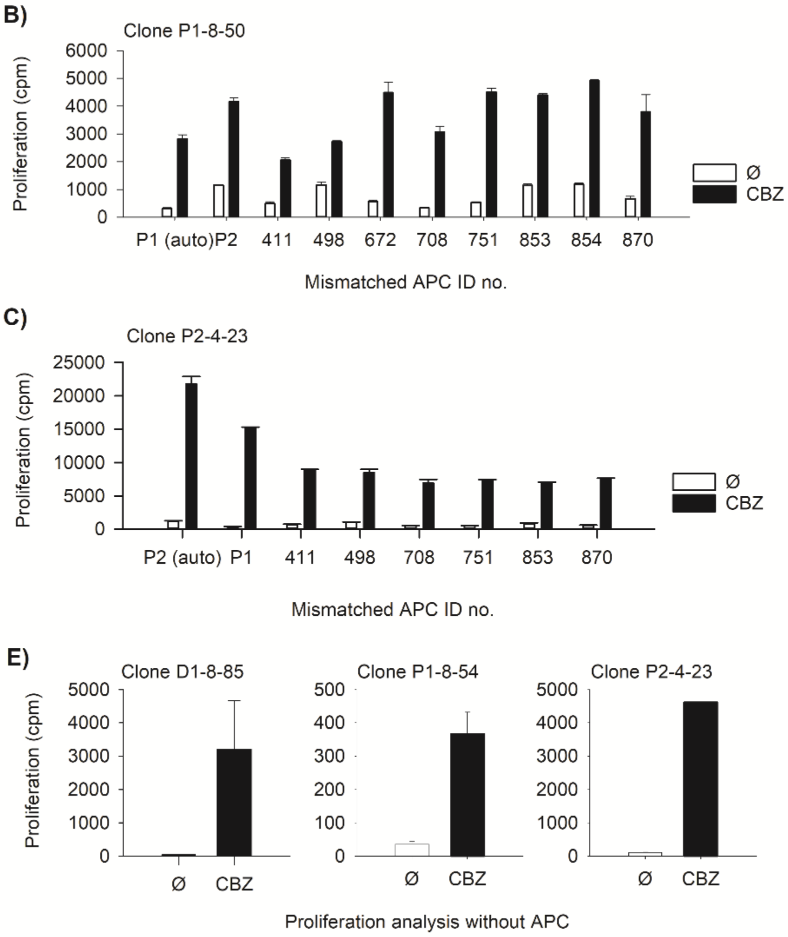

D)

APC ID no	*HLA-DRB1 genotype*	
D1	*15:01:01G	*15:02:01G
P1	*03:01	*04:04
P2	*03:01	*07:01
411	*07:01:01G	*10:01:01
465	*07:01:01G	*15:01:01G
498	*13:01:01G	*14:01:01G
560	*01:01:01G	*07:01:01G
672	*01:01:01G	*13:01:01G
708	*11:01:01G	*13:01:01G
751	*01:02:01	*07:01:01G
790	*04:05:01	*15:01:01G
802	*01:01:01G	*07:01:01G
816	*03:01:01G	*04:04:01
853	*04:01:01	*07:01:01G
854	*04:05:01	*07:01:01G
867	*11:04:01	*12:01:01G
870	*07:01:01G	*15:01:01G
976	*07:01:01G	*15:01:01G

Supplementary Figure S3. HLA mismatch analyses were unable to determine restricted HLA allele in carbamazepine (CBZ)-responsive T-cell clones with self-presenting activity. (A-D) HLA-DR-restricted CBZ-responsive T-cell clones from Donor D1 **(A)**, Patient P1 **(B)**, and Patient P2 **(C)** were tested against a panel of antigen-presenting cells (APC) with different HLA genotypes **(D)**. T-cell clones (5×10^4/well) were cultured for 48 hours at 37ºC, 5% CO_2 in duplicate with EBV-transformed B-cells (1×10^4/well) in the presence and absence of CBZ (25µg/mL). The proliferation activity was determined using [^{3}H]-thymidine incorporation assay. A representative clone was shown in each case. The results were uninterpretable due to the presence of CBZ-mediated response regardless of APCs. **(E)** The T-cell clones was shown to be responding even in the absence of APC, indicating self-presenting activity of the clones. Ø: no drug control.
